# A 25‐Year Nationwide Analysis of Mortality Trends due to Intentional Self‐Harm by Hanging, Strangulation, and Suffocation

**DOI:** 10.1002/brb3.71838

**Published:** 2026-10-16

**Authors:** Sarim Hassan Shahab, Saad Ahmed Idrees, Siddique Ahmed, Muhammad Ahmed, Hafsa Rafique, Muddassir Khalid, Fred Segawa

**Affiliations:** ^1^ Department of Psychiatry Nishtar Medical University Multan Pakistan; ^2^ Department of Psychiatry Khyber Medical College Peshawar Pakistan; ^3^ Department of Epidemiology FMH College of Medicine and Dentistry Lahore Pakistan; ^4^ Department of Public Health Makerere University College of Health Sciences, Kampala Central Region Uganda

**Keywords:** hanging, intentional self‐harm, suffocation, suicide burden, strangulation, trends

## Abstract

**Introduction:**

Suffocation, strangulation, and hanging are among the deadly forms of intentional self‐harm, which is a serious global public health issue. These types of self‐harm frequently have high fatality rates and greatly increase the United States’ suicide burden. Analyzing trends over time is essential for noticing evolving patterns, directing preventative measures, and developing successful policy interventions.

**Methods:**

To assess mortality data from the Centers for Disease Control and Prevention Wide‐ranging Online Data for Epidemiologic Research (CDC WONDER), ICD‐10 codes for intentional self‐harm by hanging, strangulation, and suffocation (X70) were utilized. Age‐adjusted mortality rates (AAMRs) were considered for analysis. Trends were assessed using the joinpoint regression software, and *p* < 0.05 was deemed statistically significant.

**Results:**

The overall AAMR per 100,000 rose from 2.41 in 1999 to 4.57 in 2023, peaking at 5.28 in 2018. Men, young adults, and Non‐Hispanic Whites had disproportionately higher AAMR, with Hawaii, South Dakota, and New Mexico showing the highest rates. Rural areas and the West and Midwest regions experienced the greatest mortality burden.

**Conclusion:**

Targeted mental health policy and resource allocation in vulnerable communities are necessary in light of these disparities.

## Graphical abstract

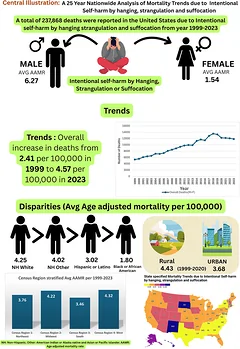

## 1 Introduction

Intentional self‐harm by hanging, strangulation, or suffocation refers to the deliberate interruption of breathing and is one of the most frequently used and one of the most lethal suicide methods in the world (Elnour and Harrison 2007). It is characterized by rapid onset of hypoxia (Gilbert et al. 2008) and requires minimal specialized equipment and a high quick‐fatality rate that only trails behind firearms (Cai et al. 2022). An estimated 700,000 people take their lives every year around the world, where suicide has a high toll among adolescents and young adults, and a large proportion of deaths occur in low‐ and middle‐income countries. Suicides in general have been on the rise in the United States over two decades, and deaths due to hanging and suffocation have been on an upward trend in various demographic groups (Baker et al. 2013). Mental disorders, previous suicide attempts, substance use, and socioeconomic stress factors, including unemployment and low educational levels of the population, are evident in systematic review findings of the Eastern Mediterranean (Zuberi et al. 2021). Moreover, family conflict, emotional distress, and lack of access to mental health care are frequent factors that correlate in content‐analysis studies from South Asia (Mahesar et al. 2024).

Intentional self‐harm through hanging and asphyxiation is a common method of suicide in many parts of the world, accounting for approximately 8% to 70% of the suicides in regions with the highest incidences found in Asia (Bachmann 2018). In population‐based studies (for example, Iran), hanging as a means of completion is close to 79%, and most victims are males, on average 33 years of age (Moftakhar et al. 2023; Joseph et al. 2022). Geographic differences can also be observed, with rural regions remaining significantly higher in suffocation suicide rates when compared to the urban ones, indicating the necessity to apply specific interventions (Pettrone and Curtin 2020). Recent epidemiological evidence indicates that hangings/suffocation suicides have steadily grown, especially among young people, women, and rural areas in the United States (Fontanella et al. 2015; Diamond et al. 2022)

Although substantial research on suicide has been done, limited recent 25‐year method‐specific joinpoint analyses incorporating 2023 data are available for suicide based on hanging, strangulation, and suffocation at the national level using mortality databases in the United States. This gap underscores the need for a comprehensive national‐level analysis of mortality trends related to suicide by hanging, strangulation, and suffocation in the United States. Thus, the goal of the research is to explore the mortality rates in intentional self‐harm by hanging, strangulation, and suffocation in the United States using the data provided by the CDC, by focusing on demographic patterns and their role in understanding counteracting public health measures.

## 2 Methods

### 2.1 Study Design and Data Source

In this descriptive study, mortality data were extracted for analysis from the Centers for Disease Control and Prevention Wide‐ranging Online Data for Epidemiologic Research (CDC WONDER), concentrating on death records spanning from 1999 to 2023 (Friede et al. 1993). The data were obtained from US death certificates using the Multiple Cause‐of‐Death (MCD) database, which includes records from all 50 states and the District of Columbia.

### 2.2 Study Population

Mortality records were identified using International Classification of Diseases, 10th Revision (ICD‐10) codes specific to intentional self‐harm by hanging, strangulation, and suffocation (X70) (World Health Organisation 1992). Deaths were identified if X70 was listed as either the underlying or a contributing cause of death, allowing for broader attribution of X70‐related mortality. This approach was selected to capture deaths in which X70 contributed to mortality, consistent with prior MCD‐based studies. A sensitivity analysis restricted to the underlying cause of death only was performed to assess the robustness of the findings. Sensitivity analysis using only the underlying cause of death produced nearly identical estimates, indicating that inclusion of contributing causes did not significantly affect the results.

### 2.3 Data Abstraction

The variables selected for analysis in the study included population size, year of death, location of death, demographics, 10‐year age groups, geographic region and states, and urban–rural classification. Demographic attributes included age, sex, and race/ethnicity; location; and setting of death. Location and setting of death were grouped into medical facilities (including outpatient, emergency department, inpatient, dead on arrival, or unknown status), home, hospice, and long‐term care facilities. Race and ethnicity were classified into categories: non‐Hispanic (NH) White, NH Black, and Hispanic or Latino. The NH American Indian or Alaskan Native, NH Asian and Pacific Islander groups, or Native Hawaiian were collectively referred to as NH Others. This information is based on data collected from death certificates and has been utilized in earlier analysis of the WONDER database (Rao et al. 2025). For analysis, we categorized participants into three broad age groups: young adults (15–44 years), middle‐aged adults (45–64 years), and older adults (≥65 years). These categories were created by combining the original age bands reported in the source data as follows: 15–24, 25–34, and 35–44 for young adults; 45–54 and 55–64 for middle‐aged adults; and 65–74, 75–84, and ≥85 for older adults. This collapsing allowed for more robust comparisons across broader age groups consistent with prior CDC WONDER database studies (Abdul Jabbar et al. 2025).

The Urban‐Rural Classification Scheme from the National Center for Health Statistics was utilized to evaluate the population based on urban areas (large metropolitan areas with a population of over 1 million and medium/small metropolitan areas with populations between 50,000 and 999,999) and rural areas (counties with populations under 50,000) according to the 2013 US Census classification (Anderson and Rosenberg 1998). Census regions were classified into Northeast, Midwest, West, and South, based on US Census Bureau definitions (Ingram et al. 2014). The overall study period covered 1999–2023. However, the availability of certain stratification variables differed within the database. State‐level and urban–rural classification data were available only through 2020, and therefore analyses stratified by these variables were restricted to the period 1999–2020. All other demographic and national‐level analyses were conducted using the full dataset through 2023. Although the CDC WONDER database offers data that are representative on a national scale, it does not include individual‐level clinical variables (such as vital signs and treatment information), which are crucial for understanding the extent of disease.

### 2.4 Statistical Analysis

To examine national trends in intentional self‐harm by hanging, strangulation, and suffocation, we calculated age‐adjusted mortality rates (AAMRs) per 100,000 population from 1999 to 2023 by year, sex, race/ethnicity, state, and urban–rural status along with 95% confidence intervals (CIs). AAMRs were calculated for overall analyses, whereas age‐specific mortality rates were reported as crude mortality rates (CMRs) within predefined age strata. National trends in AAMRs and CMRs were evaluated using the Joinpoint Regression Program version 5.4.0 (National Cancer Institute), which identifies statistically significant changes in trends over time using log‐linear models and calculates the annual percentage change (APC) and 95% CIs. *p* value lower than 0.05 was deemed statistically significant.

The methodological approach was consistent with previously published analyses, utilizing APC and AAPC to evaluate temporal trends (Imran et al. 2026).

### 2.5 Ethical Considerations

This study made use of information sourced from the CDC WONDER database, which is a dataset accessible to the public. As a result, approval from the institutional review board and informed consent are not applicable. The study followed the STROBE (Strengthening the Reporting of Observational Studies in Epidemiology) guidelines to guarantee appropriate reporting of observational studies.

## 3 Results

A total of 237,868 deaths occurred due to intentional self‐harm by hanging, strangulation, and suffocation. Out of these, 59.82% (*n* = 142,315) happened at the home of the decedent, 14.91% (*n* = 35,483) at medical facilities, 24.49% (*n* = 58,252) in other locations, and 0.37% (*n* = 883) in nursing home/long‐term care facilities (Table S1).

### 3.1 Annual Trends for Overall Mortality–Related AAMR

Over the study period from 1999 to 2023, the AAMR showed significant changes with distinct trend periods.

The AAMR was 2.41 per 100,000 in 1999 and rose significantly to 5.28 per 100,000 by 2018. The progression of the AAMR through the years has shown a uniform upward trend, having an annual percent change (APC) rate of 4.0 (95% CI: 3.73 to 4.25; *p* < 0.001) from 1999 to 2018. This signifies a long‐term upward trend in mortality rates over the course of the 19 years reviewed. From 2018 to 2023, however, this trend reversed as the AAMR dropped to 4.57, signifying a decrease from 5.28 to 4.57 per 100,000, such that the APC for this 5‐year period was −2.55 (95% CI: −4.10 to −0.98; *p* = 0.003). This demonstrates a considerable decrease in mortality rates over this 5‐year period. The overall average annual percent change (AAPC) in mortality across the study period from 1999 to 2023 was reported to be 2.59 (95% CI: 2.22 to 2.96; *p* < 0.001). This reflects the overall upward trajectory despite the recent decline (Figure 1; Table S2).

**FIGURE 1:**
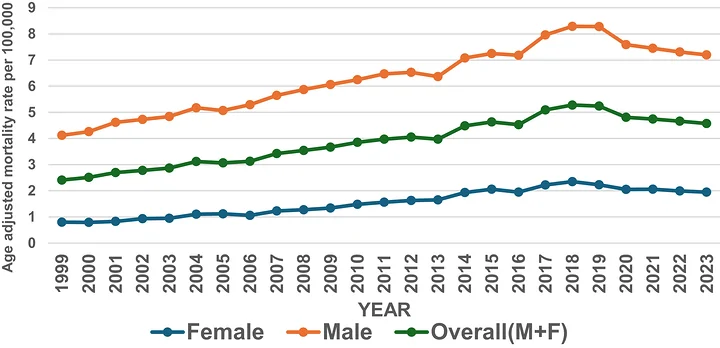
Overall trends and gender disparities in mortality due to intentional self‐harm by hanging, strangulation, and suffocation in the United States, 1999–2023.

### 3.2 AAMR Stratified by Sex

Men had a consistently higher AAMR than women during the entire study period. In men, the AAMR rose from 4.12 in 1999 to 8.29 in 2018 (APC: 3.52, 95% CI: 3.25 to 3.78; *p* < 0.001). From 2018 to 2023, the AAMR decreased significantly to 7.20 (APC: −2.49, 95% CI: −4.04 to −0.92; *p* = 0.004).

In women, the AAMR increased from 0.80 in 1999 to 2.35 in 2018 (APC: 6.00, 95% CI: 5.60 to 6.40; *p* < 0.001). From 2018 to 2023, the AAMR decreased to 1.95 (APC: −3.64, 95% CI: −5.82 to −1.42; *p* = 0.001).

From 1999 to 2023, men had an AAPC of 2.23 (95% CI: 1.86 to 2.61; *p* < 0.001), while women demonstrated an AAPC of 3.87 (95% CI: 3.36 to 4.39; *p* < 0.001) (Figure 1; Table S2).

### 3.3 AAMR Stratified by Race/Ethnicity

When stratified by race/ethnicity, AAMRs showed distinct patterns across racial and ethnic groups over the study period from 1999 to 2023.

The highest AAMR was observed in NH White adults. The AAMR rose from 2.56 in 1999 to 6.41 in 2018 (APC: 4.65, 95% CI: 4.35 to 4.95; *p* < 0.001), followed by a decrease to 5.35 in 2023 (APC: −3.76, 95% CI: −5.55 to −1.95; *p* = 0.002). Over the entire study period, NH White adults showed an AAPC of 2.84 (95% CI: 1.41 to 2.91; *p* < 0.001).

For the Other NH race category, which includes American Indian or Alaska Natives, Asian, Pacific Islander, and Native Hawaiian adults, the AAMR increased from 3.71 in 1999 to 5.19 in 2021 (APC: 2.06, 95% CI: 1.65 to 2.47; *p* < 0.001), followed by a decrease to 4.28 in 2023 (APC: −9.07, 95% CI: −20.69 to 3.73; *p* = 0.17). Over the entire study period, Other NH race adults demonstrated a non‐significant AAPC of 1.08 (95% CI: −0.009 to 2.19; *p* = 0.051).

In the Hispanic or Latino population, the AAMR rose from 2.26 in 1999 to 2.75 in 2013 (APC: 1.68, 95% CI: 1.05 to 2.32; *p* < 0.001), followed by a continued increase to 3.88 in 2018 (APC: 5.56, 95% CI: 2.58 to 8.63; *p* = 0.001), and then a non‐significant decrease to 3.86 in 2023 (APC: −0.17, 95% CI: −1.61 to 1.99; *p* = 0.84). Over the entire study period, Hispanic or Latino adults showed an AAPC of 2.16 (95% CI: 1.41 to 2.92; *p* < 0.001).

The lowest AAMR was observed among NH Black or African American adults, consistently remaining low, increasing only from 1.27 to 2.40 from 1999 to 2023. Over the entire study period, NH Black or African American adults demonstrated an AAPC of 2.39 (95% CI: 0.79 to 4.00; *p* = 0.003).

Overall, the highest AAMR was observed in NH White adults, followed by NH Others, Hispanic or Latino, and NH Black or African American, respectively (Figure 2; Table S3).

**FIGURE 2:**
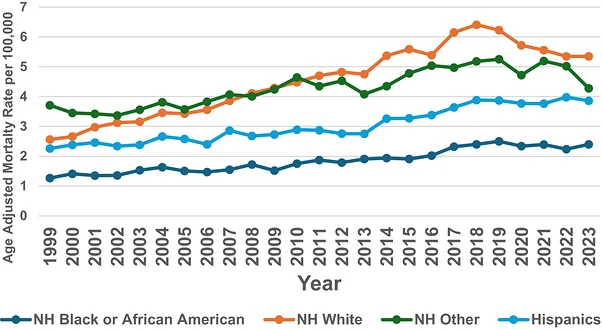
Racial disparities in mortality due to intentional self‐harm by hanging, strangulation, or suffocation in the United States, 1999–2023.

### 3.4 AAMR Stratified by State

Hawaii had the highest Average Annual Miles Run (AAMR) at 7.55, followed by South Dakota (6.72), New Mexico (6.59), Alaska (6.41), and Colorado (5.50). The District of Columbia (AAMR 2.48) had the lowest AAMR, followed by Louisiana (2.97), Mississippi (2.77), Georgia (2.87), and Alabama (3.01). Therefore, the highest‐burden states had AAMRs that were between 2 and 3 times greater than the lowest‐burden states (Figure 3; Table S4).

**FIGURE 3:**
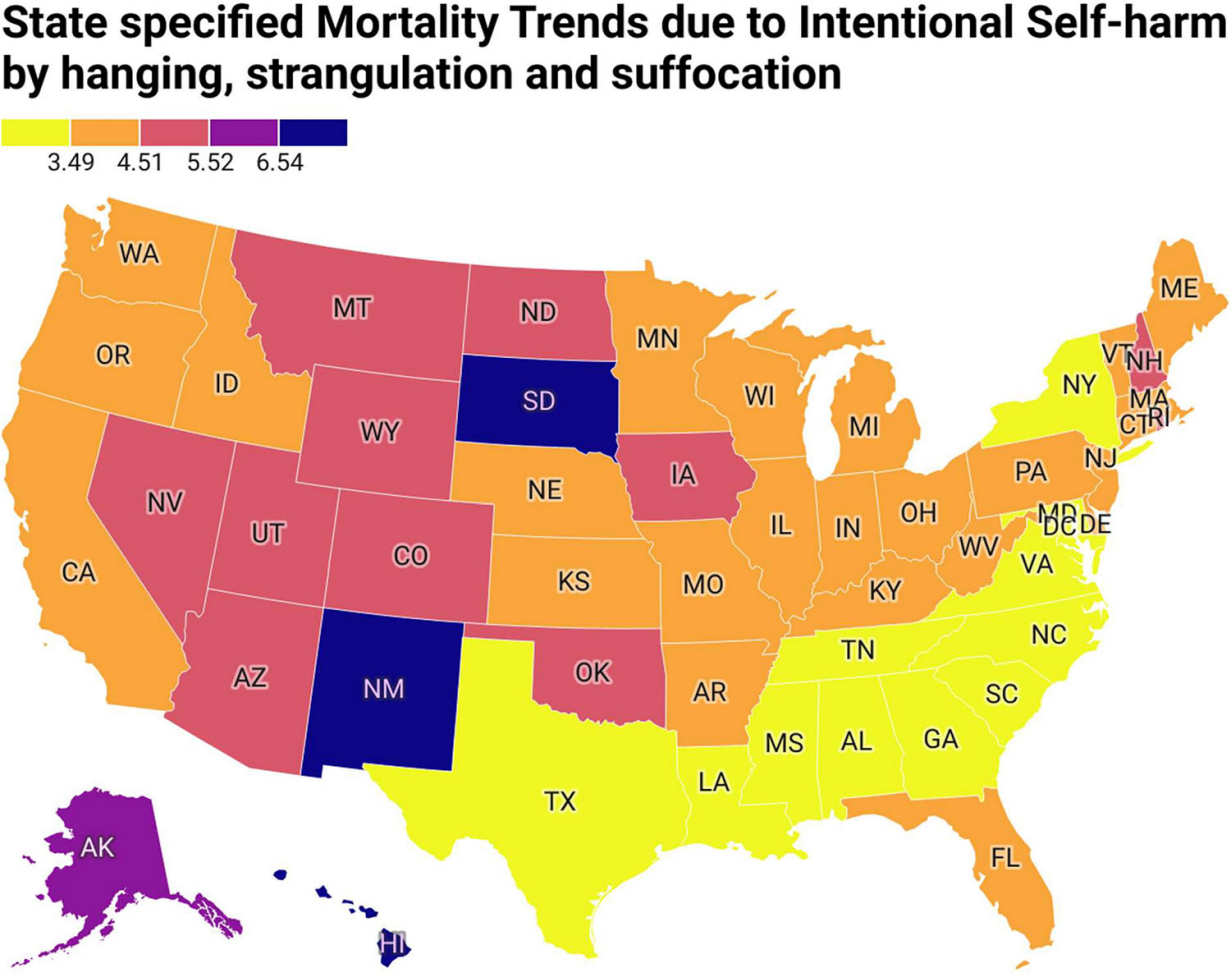
State‐wise age‐adjusted mortality rates (AAMRs) due to intentional self‐harm by hanging, strangulation, or suffocation in the United States, 1999–2020.

### 3.5 AAMR Stratified by Urban–Rural

In urban areas (metropolitan regions), the AAMR increased from 2.42 in 1999 to 5.05 in 2018 (APC: 3.73, 95% CI: 3.44 to 4.02; *p* < 0.001), followed by a decrease to 4.50 in 2020 (APC: −3.72, 95% CI: −10.90 to 4.05; *p* = 0.32). Throughout the study period, these areas had an AAPC of 2.99 (95% CI: 2.25 to 3.74; *p* < 0.001). This reflects a consistent upward trajectory with a recent decline.

In rural areas (nonmetropolitan regions), the AAMR increased markedly from 2.42 in 1999 to 7.03 in 2020. Joinpoint regression detected no significant joinpoints, so a single linear trend was fitted, yielding an AAPC of 5.51 (95% CI: 5.21 to 5.81; *p* < 0.001) (Table S5).

### 3.6 Census Region–Level Variation in AAMR

Census Region 1: Northeast: Average mortality levels showed moderate increases initially, with the average annual mortality rate (AAMR) rising from 2.55 (1999) to 4.99 (2017), with an APC of 3.83 (95% CI: 3.33–4.32) over this period (*p* < 0.001). The AAMR level then dropped to 4.13 (2023) for an APC of −3.25 (95% CI: −5.34 to 1.12) (*p* = 0.005). The AAPC for this region over the entire study period of 1999–2023 was 2.10 (95% CI: 1.39–2.83) (*p* < 0.001).

Census Region 2: Midwest: The Midwest Region had significant increases in average mortality levels, with an AAMR of 2.54 (1999) and rising to 5.82 (2018), with an APC of 4.30 (95% CI: 3.97–4.64) over this time period (*p* < 0.001), then declining to AAMR of 5.11 (2023) with an APC of ‐2.61 (95% CI: −4.58 to 0.60) (*p* = 0.014). The AAPC for the Midwest Region during the entire study period of 1999–2023 was 2.62 (95% CI: 2.35–3.30) (*p* < 0.001).

Census Region 3: South: Average mortality levels in the South Region displayed unique patterns with several phases of increase. An AAMR of 2.02 (1999) increased to 3.43 (2007) with an APC of 5.32 (95% CI: 4.19–6.45) (*p* < 0.001), followed by a modest increase to 4.81 (2013) (APC: 2.39 (95% CI: 0.62–4.20) (*p* = 0.012). The AAMR rose to 4.80 (2018) at an APC of 6.12 (95% CI: 3.72–8.57) (*p* = 0.00007) with a decline in AAMR to 4.22 (2023) for the final APC of −2.68 (95% CI: −4.15 to 1.19) (*p* = 0.002). The AAPC for the South Region over the study period of 1999–2023 was 3.03 (95% CI: 2.28–3.79) (*p* < 0.001).

Census Region 4: West: This region had notable mortality progression. The AAMR increased from 2.83 in 1999 to 5.88 in 2018 (APC: 3.74, 95% CI: 3.40 to 4.09; *p* < 0.001), followed by a decline to 4.96 in 2023 (APC: −2.47, 95% CI: −4.42 to −0.47; *p* = 0.018). Over the entire study period, the Western region showed an AAPC of 2.42 (95% CI: 1.94 to 2.90; *p* < 0.001) (Figure 4; Table S6).

**FIGURE 4:**
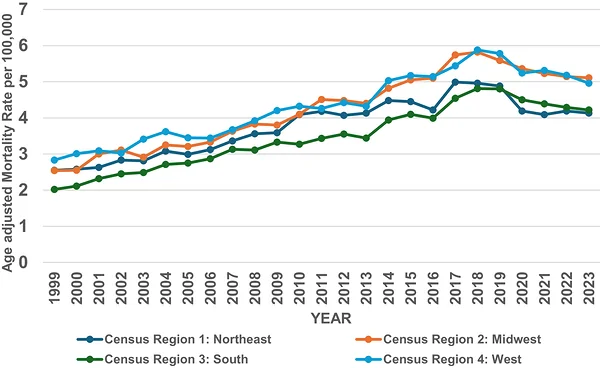
Regional disparities in age‐adjusted mortality rates (AAMR) due to intentional self‐harm by hanging, strangulation, or suffocation in the United States, 1999–2023.

### 3.7 Age‐Stratified AAMR

Young adults (15–44 years) experienced the highest mortality. The CMR increased from 2.95 in 1999 to 6.20 in 2019 (APC = 3.66, 95% CI: 3.38 to 3.93, *p* < 0.000001), followed by a decline to 5.30 in 2023 (APC = −3.97, 95% CI: −6.51 to 1.36, *p* = 0.005). The overall trend from 1999 to 2023 had an AAPC of 2.34 (95% CI: 1.86–2.83, *p* < 0.000001).

Middle‐aged adults (45–64 years) showed moderate mortality. The CMR increased from 1.77 in 1999 to 4.62 in 2015 (APC = 6.06, 95% CI: 5.14 to −6.99, *p* < 0.000001), then slightly declined to 4.39 in 2023 (APC = −0.92, 95% CI: −2.67 to 0.86, *p* = 0.292). The overall AAPC from 1999 to 2023 was 3.68 (95% CI: 2.87 to 4.49, *p* < 0.000001).

Older adults (65+ years) experienced the lowest CMRs. The rate decreased slightly from 1.74 in 1999 to 1.50 in 2009 (APC = −1.11, 95% CI: −2.22 to 0.01, *p* = 0.052), increased to 1.94 in 2014 (APC = 4.83, 95% CI: 0.67 to 9.16, *p* = 0.025), and plateaued through 2023 (APC = 0.05, 95% CI: −0.99 to 1.11, *p* = 0.921). The overall AAPC was 0.54 (95% CI: −0.43 to 1.52, *p* = 0.278).

This shows that young adults carried the highest mortality burden, middle‐aged adults experienced moderate increases over time, and older adults had the lowest mortality with minor fluctuations and a period of rise in 2009–2014 (Figure 5; Table S7).

**FIGURE 5:**
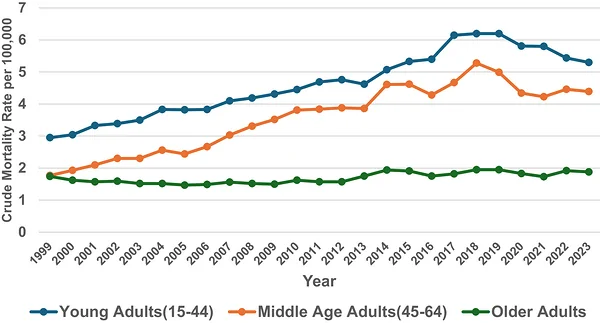
Trends in crude mortality rates (CMRs) due to intentional self‐harm by hanging, strangulation, or suffocation among different age groups in the United States, 1999–2023.

## 4 Discussion

This nationwide study of intentional self‐harm by hanging identifies important temporal, gender, racial/ethnic, and geographic patterns, accounting for 237,868 deaths. Significant mortality trends were observed. Specifically, the AAMR doubled from 2.41 to 5.28 per 100,000 between 1999 and 2018. Subsequently, the rate declined to 4.57 per 100,000 between 2018 and 2023. Examining gender differences, men consistently exhibited higher mortality rates than women, although women experienced a proportionally greater increase during the study period. Racial and ethnic disparities were evident: NH White adults had the highest rates, while NH Black adults consistently demonstrated the lowest. Geographically, analyses highlighted elevated rates in rural areas and in the Midwest and Western census regions, with lower mortality in the South and Northeast. These findings are consistent with broader epidemiological trends in suicide mortality and highlight demographic and regional disparities that require targeted public health interventions.

The AAMR increased for nearly two decades, reaching an inflection point before declining in recent years. Despite this decline, the overall trend across the study period indicates a persistent upward trajectory. The consistent rise in mortality until 2018 parallels broader United States and global suicide trends reported in the early twenty‐first century (Hedegaard et al. 2018). The decline observed after 2018 is notable and may be attributable to suicide prevention initiatives, including crisis helplines, increased mental health awareness, and community‐based interventions. Additionally, the reduction in mortality rates may be associated with greater emphasis on prevention programs, heightened awareness, increased self‐reporting, and effective crisis interventions, particularly during the COVID‐19 era when self‐reporting practices evolved. While this pattern may reflect the impact of suicide prevention initiatives, these interpretations are hypothesis‐generating rather than causal. The observed decline may also be influenced by alternative factors, including changes in death reporting practices, potential misclassification of cause of death, or delayed mortality effects related to broader societal disruptions. This reversal is further supported by findings from Pirkis et al. (2021), who reported that suicide rates in many high‐income settings during the early pandemic period were stable or lower than projected based on pre‐pandemic trends (Pirkis et al. 2021). Similar patterns have been observed in other studies of early‐pandemic data, reinforcing the plausibility of a genuine shift in the trajectory (Gunnell et al. 2020).

AAMRs remained higher among men than women throughout the study period. Both groups experienced increasing rates until 2018, followed by a subsequent decline. The mortality rate among women, however, increased more sharply, suggesting a notable change in the trajectory for this group. Persistently elevated mortality rates among men are consistent with the gender paradox in suicide, in which males have higher suicide mortality despite females reporting more ideation and attempts. This difference is primarily attributed to the use of more lethal methods by men and to cultural norms that discourage emotional expression and help‐seeking, often linked to traditional masculinity (Värnik 2012; Schrijvers et al. 2012). The sharper increase in mortality among women may reflect significant sociocultural changes, evolving gender roles, increased reporting, and greater access to means. These trends are consistent with North American data showing rising rates of suicidal ideation and nonfatal attempts among women, while overall mortality remains lower (Carretta et al. 2023).

Across racial/ethnic groups, suicide mortality was highest among NH White adults, followed by other NH races and Hispanic adults, while rates among NH Black adults remained consistently lowest. Although trends varied, most groups showed rising rates until the late 2010s, followed by recent declines. The highest mortality rates among NH White adults match previous studies that show hanging is more common among White men (Bridge et al. 2018). Higher mortality rates observed among NH White adults may reflect differences in method distribution, access to means, and sociocultural factors influencing suicide behavior, rather than individual or group‐specific intent. In contrast, NH Black adults had the lowest rates during the study period. However, new research indicates that suicide risk is increasing among Black youth, which might not be fully reflected in long‐term data specific to methods (Lindsey et al. 2019). Hispanic adults faced a moderate risk, while the NH Other category, which includes American Indian/Alaska Native (AI/AN) and Asian/Pacific Islander groups, showed higher rates in some years. This corresponds with evidence of notably high suicide rates among AI/AN populations (Leavitt et al. 2018). These issues stem from historical trauma, socioeconomic challenges, and limited access to healthcare. These disparities highlight the need for culturally specific suicide prevention programs.

Western and northern states, such as Hawaii, Alaska, South Dakota, New Mexico, and Colorado, experienced the highest mortality burden. These states reported rates two to three times greater than those observed in the District of Columbia, Louisiana, Mississippi, Georgia, and Alabama. Geographic disparities were also evident between urban and rural areas. Urban regions exhibited a steady increase in mortality rates, followed by a recent plateau. In contrast, rural areas experienced a sharper and sustained rise, ultimately surpassing urban mortality rates. This pronounced geographic variation in age‐adjusted suicide rates reflects a broader national pattern of rural–urban disparities. Over the past two decades, rural areas have experienced significantly steeper increases in suicide rates compared to metropolitan regions, which has amplified existing inequities (CDC. Rural Health 2024; Pettrone and Curtin 2020)

Rural youth face significantly elevated suicide risks, with rates nearly double those of their urban counterparts. This disparity underscores persistent challenges in mental health service access and the influence of cultural factors such as stigma and social isolation. Methodological data further indicate that rural areas have experienced disproportionately larger increases in suicide rates since the early 2000s, particularly among male and middle‐aged populations (Ivey‐Stephenson et al. 2017).

The Midwest and West saw the largest increases in AAMRs, peaking around 2018 before experiencing slight declines. This trend is similar to the national suicide patterns in these regions (Martínez‐Alés et al. 2022; Zalsman et al. 2017). This may point to the benefits of strong healthcare systems and focused prevention efforts. In the South, mortality rates followed a more complicated pattern with several ups and downs before a recent decline, but it still had the highest long‐term growth among all regions. Although the Northeast has substantial healthcare infrastructure, the decline in AAMR observed after 2017 may be associated with regional prevention efforts or other contextual factors; however, this descriptive analysis cannot establish causality.

The increased mortality from suicide over the long term may be consistent with economic and structural stressors. Factors such as economic instability, unemployment, and substance use disorders, particularly those related to the opioid epidemic, may have disproportionately affected rural and socioeconomically disadvantaged populations, overlapping with regions that demonstrated higher mortality in this study. Substance use can increase impulsivity, worsen mental health, and thereby elevate suicide risk and influence method selection. Moreover, reduced access to firearms in certain settings may result in method substitution toward other highly lethal means such as hanging or suffocation. Media coverage and the widespread dissemination of suicide‐related information through digital platforms may further influence method trends via normalization and imitation effects. Although these factors cannot be directly assessed in this analysis, they may collectively help explain temporal trends observed prior to 2018.

These differences highlight the need for public health strategies that are tailored to each region's unique demographics and systems

### 4.1 Strengths and Limitations

This study utilized the CDC WONDER database, which provides 25 years of national mortality data. This extended timeframe enabled a comprehensive analysis of temporal, demographic, and geographic trends. Several limitations should be acknowledged. Since state‐wise and urban versus rural data were unavailable for years 2021–2023, we kept the data till 2020. Mortality data are subject to potential misclassification or underreporting due to reliance on death certificate coding. The exclusive focus on deaths by hanging excludes other suicide methods, thereby limiting the generalizability of findings to overall suicide mortality. Additionally, CDC WONDER suppresses data for small cell counts, which may reduce accuracy for smaller subgroups. Future studies using disaggregated racial and ethnic data (especially for the NH Other subgroup) are needed to better characterize suicide mortality patterns within these populations. Similarly, the absence of socioeconomic factors must also be considered as a limitation since they are an important determinant of mortality.

### 4.2 Future Directions

The results underscore the necessity for targeted suicide prevention initiatives. Gender‐specific strategies should address the persistently high risk among men and the increasing mortality among women. Racial and ethnic disparities necessitate culturally tailored interventions, particularly for American Indian/Alaska Native populations and Black youth. The persistent rural–urban divide in suicide mortality highlights the importance of expanding mental health service access in rural areas through telehealth, mobile crisis teams, and enhanced community outreach. Collaboration between policymakers and healthcare providers is essential to implement evidence‐based interventions, such as restricting access to lethal means, increasing mental health literacy, and promoting equitable access to care.

## 5 Conclusion

Our study shows that suicide by hanging in the United States has increased steadily for nearly two decades. The rate has been observed to decline in recent years. This shift is encouraging, but the persistent gaps observed, higher rates among men, NH White adults, and those living in rural areas, make it clear that progress has not addressed disparities. The steep incline in mortality rates among women and the new risks for minority groups point toward changing sociocultural aspects that require attention. These findings remind us that suicide is influenced not only by personal struggles but also by social, cultural, and structural factors. The modest decline in rates after 2018 offers hope that progress is possible, but sustaining and accelerating this trend will require ongoing commitment, innovation, and collaboration across healthcare, community, and policy sectors. Ultimately, reducing suicide by hanging is inseparable from the broader goal of reducing inequities and promoting mental health equity for all populations.

## Author Contributions

**Sarim Hassan Shahab**: conceptualization, methodology, software, data curation, formal analysis, writing – original draft, writing – review and editing, visualization, project administration. **Saad Ahmed Idrees**: methodology, data curation, formal analysis, validation, writing – review and editing. **Siddique Ahmed**: investigation, supervision, formal analysis, data curation. **Muhammad Ahmed**: data curation, formal analysis, methodology, validation. **Hafsa Rafique**: formal analysis, data curation, writing – review and editing. **Muddassir Khalid**: supervision, methodology, formal analysis, conceptualization. **Fred Segawa**: conceptualization, supervision, project administration, validation, writing – review and editing.

## Funding

The authors have nothing to report.

## Conflicts of Interest

The authors declare no conflicts of interest.

## Ethics Statement

This study used anonymized, publicly available data from the Centers for Disease Control and Prevention Wide‐ranging Online Data for Epidemiologic Research (CDC WONDER). Ethical approval and patient consent were therefore not required.

## Supporting information




**Supporting Information**: brb371838‐sup‐0001‐SuppMat.docx

## Data Availability

The data relevant to this work are publicly available from the CDC WONDER Database website.
