# Supplementary material for: A 25‐Year Nationwide Analysis of Mortality Trends due to Intentional Self‐Harm by Hanging, Strangulation, and Suffocation
Source: Brain Behav. 2026 Oct 16;16(10):e71838. doi: 10.1002/brb3.71838 (PMC13673224; doi:10.1002/brb3.71838)
Supplement: Supplementary file 1 [file BRB3-16-e71838-s001.docx]

**SUPPLEMENTARY TABLES**

**Rising Suicide Mortality in United States by Hanging, Strangulation, and Suffocation: A Retrospective Analysis**

**Table Legends:**

## SUPPLEMENT TABLE 1. PLACE OF DEATH (1999-2023)

## SUPPLEMENT TABLE 2. Overall and Gender wise AAMRs with upper and lower confidence interval (CI) values (1999-2023)

## SUPPLEMENT TABLE 3. Race stratified AAMRs with 95% upper and lower Confidence interval(CI) values (1999-2023)

## SUPPLEMENT TABLE 4. State wise AAMRs with upper and lower confidence interval (CI) values (1999-2020)

## SUPPLEMENT TABLE 5. Urban and rural wise AAMRs with upper and lower confidence interval (CI) values (1999-2020)

## SUPPLEMENT TABLE 6. Census region wise AAMRs with upper and lower confidence interval (CI) values (1999-2023)

## SUPPLEMENT TABLE 7. Age group wise AAMRs with upper and lower confidence interval (CI) values (1999-2023)

**Tables:**

## SUPPLEMENT TABLE 1. PLACE OF DEATH (1999-2023)

| **PLACE Of Death** | **Number of Deaths** | **Percentage of total** |
| --- | --- | --- |
| Medical Facilities | 35483 | 14.91% |
| Descendent's Home | 142315 | 59.82% |
| Nursing Home | 883 | 0.37% |
| Hospice facility | 231 | 0.10% |
| Other | 58252 | 24.49% |
| Place of death unknown | 704 | 0.30% |
| TOTAL | **237868** | 100% |

## SUPPLEMENT TABLE 2. Overall and Gender wise AAMRs with upper and lower confidence interval (CI) values (1999-2023)

| **Year** | **Sex** | **AAMR(95% CI)** | **Sex** | **AAMR(95% CI)** | **Sex(M+F)** | **AAMR(95% CI)** |
| --- | --- | --- | --- | --- | --- | --- |
| 1999 | Female | 0.8 (0.75–0.85) | Male | 4.12 (3.99–4.24) | Overall | 2.41 (2.35–2.48) |
| 2000 | Female | 0.79 (0.74–0.85) | Male | 4.26 (4.13–4.38) | Overall | 2.51 (2.44–2.57) |
| 2001 | Female | 0.83 (0.78–0.88) | Male | 4.62 (4.5–4.75) | Overall | 2.7 (2.63–2.76) |
| 2002 | Female | 0.93 (0.88–0.99) | Male | 4.73 (4.6–4.86) | Overall | 2.78 (2.71–2.85) |
| 2003 | Female | 0.95 (0.89–1.01) | Male | 4.84 (4.71–4.97) | Overall | 2.87 (2.8–2.94) |
| 2004 | Female | 1.1 (1.04–1.16) | Male | 5.17 (5.03–5.3) | Overall | 3.12 (3.04–3.19) |
| 2005 | Female | 1.12 (1.06–1.18) | Male | 5.07 (4.94–5.2) | Overall | 3.07 (3–3.14) |
| 2006 | Female | 1.06 (1–1.12) | Male | 5.29 (5.16–5.42) | Overall | 3.13 (3.06–3.21) |
| 2007 | Female | 1.23 (1.17–1.29) | Male | 5.65 (5.51–5.79) | Overall | 3.42 (3.34–3.49) |
| 2008 | Female | 1.27 (1.21–1.34) | Male | 5.87 (5.73–6.01) | Overall | 3.54 (3.46–3.62) |
| 2009 | Female | 1.34 (1.27–1.4) | Male | 6.06 (5.92–6.2) | Overall | 3.67 (3.6–3.75) |
| 2010 | Female | 1.48 (1.41–1.55) | Male | 6.25 (6.1–6.39) | Overall | 3.85 (3.77–3.92) |
| 2011 | Female | 1.56 (1.49–1.63) | Male | 6.47 (6.33–6.62) | Overall | 3.97 (3.89–4.05) |
| 2012 | Female | 1.63 (1.56–1.7) | Male | 6.53 (6.38–6.67) | Overall | 4.05 (3.97–4.13) |
| 2013 | Female | 1.65 (1.58–1.73) | Male | 6.37 (6.22–6.51) | Overall | 3.97 (3.89–4.05) |
| 2014 | Female | 1.93 (1.85–2.01) | Male | 7.08 (6.93–7.23) | Overall | 4.48 (4.4–4.56) |
| 2015 | Female | 2.06 (1.98–2.14) | Male | 7.25 (7.1–7.41) | Overall | 4.63 (4.54–4.71) |
| 2016 | Female | 1.95 (1.87–2.03) | Male | 7.18 (7.03–7.34) | Overall | 4.53 (4.44–4.61) |
| 2017 | Female | 2.22 (2.14–2.31) | Male | 7.96 (7.8–8.12) | Overall | 5.09 (5–5.18) |
| 2018 | Female | 2.35 (2.26–2.44) | Male | 8.29 (8.12–8.45) | Overall | 5.28 (5.19–5.37) |
| 2019 | Female | 2.23 (2.14–2.31) | Male | 8.28 (8.11–8.44) | Overall | 5.24 (5.14–5.33) |
| 2020 | Female | 2.05 (1.97–2.13) | Male | 7.59 (7.44–7.75) | Overall | 4.81 (4.72–4.9) |
| 2021 | Female | 2.06 (1.98–2.14) | Male | 7.45 (7.29–7.6) | Overall | 4.74 (4.65–4.82) |
| 2022 | Female | 1.99 (1.91–2.06) | Male | 7.31 (7.16–7.46) | Overall | 4.66 (4.57–4.74) |
| 2023 | Female | 1.95 (1.87–2.03) | Male | 7.2 (7.05–7.35) | Overall | 4.57 (4.49–4.65) |

## SUPPLEMENT TABLE 3. Race stratified AAMRs with 95% upper and lower Confidence interval(CI) values (1999-2023)

| **Year** | **NH Black or African American** | **NH White** | **NH Other** | **Hispanic or Latino** |
| --- | --- | --- | --- | --- |
| 1999 | 1.27 (1.13–1.40) | 2.56 (2.48–2.64) | 3.71 (3.30–4.11) | 2.26 (2.04–2.48) |
| 2000 | 1.41 (1.26–1.55) | 2.66 (2.58–2.74) | 3.45 (3.07–3.83) | 2.38 (2.16–2.59) |
| 2001 | 1.35 (1.21–1.49) | 2.97 (2.89–3.06) | 3.42 (3.05–3.79) | 2.46 (2.25–2.68) |
| 2002 | 1.36 (1.22–1.50) | 3.12 (3.03–3.21) | 3.37 (3.01–3.72) | 2.34 (2.14–2.54) |
| 2003 | 1.53 (1.38–1.67) | 3.16 (3.07–3.25) | 3.56 (3.21–3.92) | 2.38 (2.19–2.57) |
| 2004 | 1.63 (1.48–1.78) | 3.46 (3.37–3.55) | 3.81 (3.45–4.17) | 2.66 (2.46–2.86) |
| 2005 | 1.51 (1.37–1.65) | 3.43 (3.34–3.52) | 3.57 (3.22–3.91) | 2.58 (2.39–2.77) |
| 2006 | 1.47 (1.34–1.61) | 3.56 (3.46–3.65) | 3.83 (3.48–4.17) | 2.40 (2.22–2.58) |
| 2007 | 1.55 (1.41–1.69) | 3.86 (3.77–3.96) | 4.07 (3.72–4.42) | 2.86 (2.67–3.06) |
| 2008 | 1.72 (1.57–1.87) | 4.11 (4.01–4.21) | 4.01 (3.67–4.35) | 2.68 (2.50–2.87) |
| 2009 | 1.52 (1.38–1.66) | 4.29 (4.19–4.40) | 4.24 (3.90–4.58) | 2.73 (2.55–2.91) |
| 2010 | 1.75 (1.60–1.90) | 4.48 (4.37–4.59) | 4.64 (4.28–4.99) | 2.89 (2.71–3.07) |
| 2011 | 1.87 (1.72–2.03) | 4.70 (4.59–4.81) | 4.35 (4.01–4.68) | 2.87 (2.69–3.05) |
| 2012 | 1.79 (1.64–1.94) | 4.82 (4.70–4.93) | 4.53 (4.20–4.87) | 2.76 (2.59–2.93) |
| 2013 | 1.91 (1.75–2.06) | 4.75 (4.64–4.86) | 4.08 (3.77–4.39) | 2.75 (2.58–2.92) |
| 2014 | 1.94 (1.79–2.09) | 5.37 (5.25–5.49) | 4.35 (4.03–4.66) | 3.26 (3.08–3.44) |
| 2015 | 1.91 (1.76–2.06) | 5.59 (5.47–5.71) | 4.78 (4.45–5.10) | 3.27 (3.09–3.44) |
| 2016 | 2.02 (1.87–2.17) ❗ | 5.39 (5.27–5.51) | 5.04 (4.71–5.37) | 3.38 (3.20–3.55) |
| 2017 | 2.32 (2.15–2.48) | 6.15 (6.02–6.28) | 4.97 (4.64–5.29) | 3.64 (3.46–3.82) |
| 2018 | 2.40 (2.23–2.56) | 6.41 (6.27–6.54) | 5.18 (4.85–5.51) | 3.88 (3.70–4.06) |
| 2019 | 2.50 (2.33–2.67) | 6.23 (6.10–6.36) | 5.25 (4.93–5.58) | 3.87 (3.69–4.05) |
| 2020 | 2.34 (2.18–2.51) | 5.72 (5.60–5.85) | 4.72 (4.41–5.02) | 3.77 (3.59–3.95) |
| 2021 | 2.39 (2.23–2.57) | 5.56 (5.44–5.68) | 5.19 (4.86–5.53) | 3.76 (3.58–3.94) |
| 2022 | 2.24 (2.08–2.41) | 5.35 (5.23–5.47) | 5.02 (4.71–5.35) | 3.98 (3.80–4.17) |
| 2023 | 2.40 (2.23–2.57) | 5.35 (5.23–5.47) | 4.28 (3.99–4.59) | 3.86 (3.69–4.04) |

## SUPPLEMENT TABLE 4. State wise AAMRs with upper and lower confidence interval (CI) values (1999-2020)

| **State** | **Population** | **Age Adjusted Rate** | **State** | **State Code** | **Deaths** | **Population** | **Age Adjusted Rate** |
| --- | --- | --- | --- | --- | --- | --- | --- |
| Alabama | 83367249 | 3.01 (2.89–3.13) | Missouri | MO | 4136 | 104566823 | 4.13 (4–4.25) |
| Alaska | 11901376 | 6.41 (5.96–6.86) | Montana | MT | 845 | 17584099 | 5.13 (4.77–5.48) |
| Arizona | 109441064 | 4.57 (4.44–4.7) | Nebraska | NE | 1240 | 31673145 | 4.04 (3.81–4.26) |
| Arkansas | 50449510 | 4.01 (3.82–4.19) | Nevada | NV | 2105 | 45962686 | 4.63 (4.43–4.83) |
| California | 647628818 | 3.72 (3.68–3.77) | New Hampshire | NH | 1064 | 23679753 | 4.66 (4.37–4.94) |
| Colorado | 88405853 | 5.5 (5.34–5.65) | New Jersey | NJ | 5575 | 155186164 | 3.62 (3.52–3.71) |
| Connecticut | 63245500 | 4.16 (4–4.32) | New Mexico | NM | 2190 | 34857965 | 6.59 (6.31–6.87) |
| Delaware | 15850878 | 3.99 (3.67–4.31) | New York | NY | 10830 | 346700222 | 3.18 (3.12–3.24) |
| District of Columbia | 11509721 | 2.48 (2.18–2.78) | North Carolina | NC | 4790 | 165466198 | 3 (2.92–3.09) |
| Florida | 341410712 | 3.88 (3.81–3.95) | North Dakota | ND | 608 | 12268337 | 5.05 (4.65–5.46) |
| Georgia | 165295457 | 2.87 (2.79–2.95) | Ohio | OH | 7757 | 204089042 | 3.97 (3.88–4.06) |
| Hawaii | 23952223 | 7.55 (7.19–7.9) | Oklahoma | OK | 2992 | 64913496 | 4.84 (4.66–5.01) |
| Idaho | 26325103 | 4.18 (3.92–4.43) | Oregon | OR | 2975 | 68301491 | 4.5 (4.33–4.66) |
| Illinois | 223189808 | 3.87 (3.79–3.95) | Pennsylvania | PA | 8654 | 227145663 | 3.99 (3.9–4.07) |
| Indiana | 112628952 | 4.11 (3.99–4.23) | Rhode Island | RI | 865 | 19179297 | 4.67 (4.35–4.98) |
| Iowa | 53662355 | 4.67 (4.48–4.86) | South Carolina | SC | 2502 | 81252788 | 3.23 (3.1–3.36) |
| Kansas | 49033965 | 4.49 (4.3–4.69) | South Dakota | SD | 917 | 14190155 | 6.72 (6.28–7.16) |
| Kentucky | 75973132 | 3.73 (3.59–3.87) | Tennessee | TN | 3691 | 111317546 | 3.47 (3.36–3.59) |
| Louisiana | 79449837 | 2.97 (2.84–3.09) | Texas | TX | 14628 | 425467465 | 3.41 (3.36–3.47) |
| Maine | 24126423 | 4.26 (3.98–4.53) | Utah | UT | 2391 | 44322506 | 5.26 (5.04–5.47) |
| Maryland | 101628036 | 3.22 (3.11–3.33) | Vermont | VT | 447 | 11355649 | 4.19 (3.79–4.58) |
| Massachusetts | 119126075 | 4.41 (4.29–4.53) | Virginia | VA | 4282 | 140567657 | 3.1 (3–3.19) |
| Michigan | 176265043 | 3.99 (3.9–4.09) | Washington | WA | 4697 | 118841566 | 3.99 (3.87–4.1) |
| Minnesota | 92944056 | 3.84 (3.72–3.97) | West Virginia | WV | 1122 | 33215920 | 3.76 (3.53–3.98) |
| Mississippi | 51045931 | 2.77 (2.62–2.92) | Wisconsin | WI | 4092 | 100048779 | 4.22 (4.09–4.35) |
|  |  |  | Wyoming | WY | 441 | 9637722 | 4.8 (4.35–5.25) |

## SUPPLEMENT TABLE 5. Urban and rural wise AAMRs with upper and lower confidence interval (CI) values (1999-2020)

| **Year** | **Area** | **AAMR(95% CI)** | **Area** | **AAMR (95% CI)** |
| --- | --- | --- | --- | --- |
| 1999 | URBAN | 2.42 (2.35–2.49) | RURAL | 2.42 (2.25–2.58) |
| 2000 | URBAN | 2.48 (2.41–2.55) | RURAL | 2.56 (2.39–2.73) |
| 2001 | URBAN | 2.71 (2.64–2.78) | RURAL | 2.77 (2.59–2.95) |
| 2002 | URBAN | 2.8 (2.72–2.87) | RURAL | 2.87 (2.69–3.05) |
| 2003 | URBAN | 2.85 (2.78–2.93) | RURAL | 2.96 (2.78–3.15) |
| 2004 | URBAN | 3.06 (2.98–3.14) | RURAL | 3.27 (3.08–3.47) |
| 2005 | URBAN | 3.08 (3–3.16) | RURAL | 3.08 (2.89–3.26) |
| 2006 | URBAN | 3.14 (3.06–3.22) | RURAL | 3.29 (3.09–3.48) |
| 2007 | URBAN | 3.4 (3.32–3.48) | RURAL | 3.54 (3.33–3.74) |
| 2008 | URBAN | 3.48 (3.4–3.56) | RURAL | 3.88 (3.67–4.09) |
| 2009 | URBAN | 3.66 (3.58–3.75) | RURAL | 3.92 (3.71–4.13) |
| 2010 | URBAN | 3.78 (3.7–3.86) | RURAL | 4.26 (4.04–4.48) |
| 2011 | URBAN | 3.86 (3.78–3.95) | RURAL | 4.76 (4.52–5) |
| 2012 | URBAN | 3.96 (3.88–4.05) | RURAL | 4.68 (4.44–4.92) |
| 2013 | URBAN | 3.86 (3.78–3.94) | RURAL | 4.86 (4.62–5.1) |
| 2014 | URBAN | 4.36 (4.28–4.45) | RURAL | 5.15 (4.9–5.4) |
| 2015 | URBAN | 4.46 (4.37–4.55) | RURAL | 5.85 (5.59–6.12) |
| 2016 | URBAN | 4.35 (4.26–4.43) | RURAL | 5.92 (5.65–6.18) |
| 2017 | URBAN | 4.87 (4.78–4.96) | RURAL | 6.58 (6.3–6.86) |
| 2018 | URBAN | 5.05 (4.96–5.15) | RURAL | 7.01 (6.72–7.3) |
| 2019 | URBAN | 4.97 (4.87–5.06) | RURAL | 6.92 (6.62–7.21) |
| 2020 | URBAN | 4.5 (4.41–4.59) | RURAL | 7.03 (6.74–7.33) |

## SUPPLEMENT TABLE 6. Census region wise AAMRs with upper and lower confidence interval (CI) values (1999-2023)

| **Census Region** | **Year** | **AAMR (95% CI)** | **Census Region** | **Year** | **AAMR (95% CI)** |
| --- | --- | --- | --- | --- | --- |
| Northeast | 1999 | 2.55 (2.39–2.7) | Midwest | 1999 | 2.54 (2.4–2.68) |
| Northeast | 2000 | 2.58 (2.43–2.73) | Midwest | 2000 | 2.55 (2.41–2.69) |
| Northeast | 2001 | 2.63 (2.47–2.78) | Midwest | 2001 | 3 (2.85–3.15) |
| Northeast | 2002 | 2.83 (2.67–2.99) | Midwest | 2002 | 3.11 (2.96–3.27) |
| Northeast | 2003 | 2.81 (2.65–2.97) | Midwest | 2003 | 2.91 (2.76–3.06) |
| Northeast | 2004 | 3.08 (2.91–3.24) | Midwest | 2004 | 3.25 (3.1–3.41) |
| Northeast | 2005 | 2.99 (2.82–3.15) | Midwest | 2005 | 3.21 (3.05–3.36) |
| Northeast | 2006 | 3.12 (2.96–3.29) | Midwest | 2006 | 3.33 (3.17–3.49) |
| Northeast | 2007 | 3.36 (3.19–3.53) | Midwest | 2007 | 3.63 (3.46–3.79) |
| Northeast | 2008 | 3.56 (3.39–3.74) | Midwest | 2008 | 3.83 (3.66–4) |
| Northeast | 2009 | 3.59 (3.41–3.77) | Midwest | 2009 | 3.8 (3.63–3.97) |
| Northeast | 2010 | 4.09 (3.9–4.28) | Midwest | 2010 | 4.1 (3.92–4.28) |
| Northeast | 2011 | 4.18 (3.99–4.38) | Midwest | 2011 | 4.51 (4.32–4.69) |
| Northeast | 2012 | 4.07 (3.88–4.26) | Midwest | 2012 | 4.48 (4.3–4.67) |
| Northeast | 2013 | 4.13 (3.94–4.32) | Midwest | 2013 | 4.4 (4.21–4.58) |
| Northeast | 2014 | 4.48 (4.28–4.68) | Midwest | 2014 | 4.82 (4.63–5.01) |
| Northeast | 2015 | 4.45 (4.25–4.64) | Midwest | 2015 | 5.05 (4.85–5.24) |
| Northeast | 2016 | 4.22 (4.03–4.41) | Midwest | 2016 | 5.1 (4.9–5.3) |
| Northeast | 2017 | 4.99 (4.78–5.21) | Midwest | 2017 | 5.74 (5.53–5.95) |
| Northeast | 2018 | 4.96 (4.75–5.17) | Midwest | 2018 | 5.82 (5.61–6.03) |
| Northeast | 2019 | 4.88 (4.67–5.09) | Midwest | 2019 | 5.59 (5.38–5.79) |
| Northeast | 2020 | 4.19 (4–4.39) | Midwest | 2020 | 5.36 (5.15–5.56) |
| Northeast | 2021 | 4.09 (3.9–4.28) | Midwest | 2021 | 5.23 (5.03–5.43) |
| Northeast | 2022 | 4.19 (4–4.39) | Midwest | 2022 | 5.14 (4.94–5.34) |
| Northeast | 2023 | 4.13 (3.94–4.32) | Midwest | 2023 | 5.11 (4.92–5.31) |
| **Census Region** | **Year** | **AAMR (95% CI)** | **Census Region** | **Year** | **AAMR (95% CI)** |
| South | 1999 | 2.02 (1.92–2.12) | West | 1999 | 2.83 (2.68–2.98) |
| South | 2000 | 2.11 (2.01–2.21) | West | 2000 | 3.01 (2.86–3.17) |
| South | 2001 | 2.32 (2.22–2.43) | West | 2001 | 3.09 (2.93–3.24) |
| South | 2002 | 2.45 (2.34–2.56) | West | 2002 | 3.03 (2.88–3.18) |
| South | 2003 | 2.49 (2.38–2.6) | West | 2003 | 3.41 (3.25–3.57) |
| South | 2004 | 2.71 (2.6–2.82) | West | 2004 | 3.62 (3.46–3.79) |
| South | 2005 | 2.75 (2.64–2.86) | West | 2005 | 3.45 (3.29–3.61) |
| South | 2006 | 2.87 (2.75–2.98) | West | 2006 | 3.44 (3.29–3.6) |
| South | 2007 | 3.13 (3.02–3.25) | West | 2007 | 3.67 (3.5–3.83) |
| South | 2008 | 3.11 (3–3.23) | West | 2008 | 3.92 (3.76–4.09) |
| South | 2009 | 3.33 (3.21–3.45) | West | 2009 | 4.2 (4.03–4.37) |
| South | 2010 | 3.27 (3.15–3.38) | West | 2010 | 4.32 (4.15–4.49) |
| South | 2011 | 3.43 (3.31–3.56) | West | 2011 | 4.26 (4.09–4.43) |
| South | 2012 | 3.55 (3.43–3.68) | West | 2012 | 4.42 (4.24–4.59) |
| South | 2013 | 3.44 (3.31–3.56) | West | 2013 | 4.32 (4.16–4.49) |
| South | 2014 | 3.94 (3.81–4.07) | West | 2014 | 5.03 (4.85–5.21) |
| South | 2015 | 4.1 (3.97–4.23) | West | 2015 | 5.17 (4.99–5.35) |
| South | 2016 | 3.99 (3.86–4.12) | West | 2016 | 5.14 (4.96–5.32) |
| South | 2017 | 4.54 (4.4–4.67) | West | 2017 | 5.44 (5.25–5.63) |
| South | 2018 | 4.81 (4.67–4.95) | West | 2018 | 5.88 (5.69–6.08) |
| South | 2019 | 4.8 (4.66–4.94) | West | 2019 | 5.78 (5.58–5.97) |
| South | 2020 | 4.5 (4.36–4.63) | West | 2020 | 5.24 (5.05–5.42) |
| South | 2021 | 4.39 (4.26–4.52) | West | 2021 | 5.31 (5.13–5.5) |
| South | 2022 | 4.29 (4.16–4.42) | West | 2022 | 5.18 (5–5.36) |
| South | 2023 | 4.22 (4.09–4.35) | West | 2023 | 4.96 (4.79–5.14) |

## SUPPLEMENT TABLE 7. Age group wise AAMRs with upper and lower confidence interval (CI) values (1999-2023)

| Age Group | Year | Crude Rate (95% CI) | Age Group | Year | Crude Rate (95% CI) | Age Group | Year | Crude Rate (95% CI) |
| --- | --- | --- | --- | --- | --- | --- | --- | --- |
| Young Adults (15-44) | 1999 | 2.95 (2.85–3.04) | Middle Age Adults (45-64) | 1999 | 1.77 (1.67–1.88) | Older Adults (65+) | 1999 | 1.74 (1.6–1.87) |
| Young Adults (15-44) | 2000 | 3.04 (2.94–3.13) | Middle Age Adults (45-64) | 2000 | 1.93 (1.82–2.04) | Older Adults (65+) | 2000 | 1.62 (1.49–1.75) |
| Young Adults (15-44) | 2001 | 3.33 (3.23–3.43) | Middle Age Adults (45-64) | 2001 | 2.1 (1.99–2.21) | Older Adults (65+) | 2001 | 1.57 (1.44–1.7) |
| Young Adults (15-44) | 2002 | 3.39 (3.29–3.49) | Middle Age Adults (45-64) | 2002 | 2.3 (2.18–2.41) | Older Adults (65+) | 2002 | 1.59 (1.46–1.72) |
| Young Adults (15-44) | 2003 | 3.5 (3.4–3.61) | Middle Age Adults (45-64) | 2003 | 2.3 (2.19–2.42) | Older Adults (65+) | 2003 | 1.52 (1.39–1.65) |
| Young Adults (15-44) | 2004 | 3.83 (3.72–3.93) | Middle Age Adults (45-64) | 2004 | 2.56 (2.44–2.68) | Older Adults (65+) | 2004 | 1.52 (1.39–1.65) |
| Young Adults (15-44) | 2005 | 3.82 (3.71–3.93) | Middle Age Adults (45-64) | 2005 | 2.44 (2.33–2.55) | Older Adults (65+) | 2005 | 1.47 (1.34–1.59) |
| Young Adults (15-44) | 2006 | 3.83 (3.72–3.94) | Middle Age Adults (45-64) | 2006 | 2.67 (2.55–2.79) | Older Adults (65+) | 2006 | 1.49 (1.37–1.62) |
| Young Adults (15-44) | 2007 | 4.1 (3.99–4.21) | Middle Age Adults (45-64) | 2007 | 3.03 (2.9–3.15) | Older Adults (65+) | 2007 | 1.56 (1.43–1.69) |
| Young Adults (15-44) | 2008 | 4.19 (4.08–4.3) | Middle Age Adults (45-64) | 2008 | 3.31 (3.18–3.43) | Older Adults (65+) | 2008 | 1.52 (1.4–1.64) |
| Young Adults (15-44) | 2009 | 4.31 (4.2–4.43) | Middle Age Adults (45-64) | 2009 | 3.52 (3.39–3.65) | Older Adults (65+) | 2009 | 1.5 (1.38–1.62) |
| Young Adults (15-44) | 2010 | 4.45 (4.33–4.57) | Middle Age Adults (45-64) | 2010 | 3.81 (3.68–3.95) | Older Adults (65+) | 2010 | 1.62 (1.5–1.75) |
| Young Adults (15-44) | 2011 | 4.69 (4.57–4.81) | Middle Age Adults (45-64) | 2011 | 3.84 (3.71–3.98) | Older Adults (65+) | 2011 | 1.57 (1.45–1.69) |
| Young Adults (15-44) | 2012 | 4.76 (4.64–4.88) | Middle Age Adults (45-64) | 2012 | 3.88 (3.75–4.02) | Older Adults (65+) | 2012 | 1.57 (1.45–1.68) |
| Young Adults (15-44) | 2013 | 4.62 (4.5–4.74) | Middle Age Adults (45-64) | 2013 | 3.86 (3.73–4) | Older Adults (65+) | 2013 | 1.75 (1.63–1.87) |
| Young Adults (15-44) | 2014 | 5.07 (4.95–5.2) | Middle Age Adults (45-64) | 2014 | 4.61 (4.46–4.76) | Older Adults (65+) | 2014 | 1.94 (1.81–2.07) |
| Young Adults (15-44) | 2015 | 5.33 (5.21–5.46) | Middle Age Adults (45-64) | 2015 | 4.62 (4.48–4.77) | Older Adults (65+) | 2015 | 1.91 (1.79–2.04) |
| Young Adults (15-44) | 2016 | 5.4 (5.28–5.53) | Middle Age Adults (45-64) | 2016 | 4.28 (4.14–4.42) | Older Adults (65+) | 2016 | 1.75 (1.64–1.87) |
| Young Adults (15-44) | 2017 | 6.15 (6.02–6.29) | Middle Age Adults (45-64) | 2017 | 4.67 (4.52–4.82) | Older Adults (65+) | 2017 | 1.82 (1.71–1.94) |
| Young Adults (15-44) | 2018 | 6.2 (6.07–6.34) | Middle Age Adults (45-64) | 2018 | 5.28 (5.13–5.44) | Older Adults (65+) | 2018 | 1.95 (1.83–2.07) |
| Young Adults (15-44) | 2019 | 6.2 (6.06–6.33) | Middle Age Adults (45-64) | 2019 | 4.99 (4.84–5.14) | Older Adults (65+) | 2019 | 1.95 (1.84–2.07) |
| Young Adults (15-44) | 2020 | 5.81 (5.68–5.94) | Middle Age Adults (45-64) | 2020 | 4.34 (4.19–4.48) | Older Adults (65+) | 2020 | 1.83 (1.72–1.95) |
| Young Adults (15-44) | 2021 | 5.8 (5.67–5.93) | Middle Age Adults (45-64) | 2021 | 4.23 (4.09–4.37) | Older Adults (65+) | 2021 | 1.73 (1.62–1.84) |
| Young Adults (15-44) | 2022 | 5.44 (5.32–5.57) | Middle Age Adults (45-64) | 2022 | 4.46 (4.31–4.6) | Older Adults (65+) | 2022 | 1.92 (1.81–2.03) |
| Young Adults (15-44) | 2023 | 5.3 (5.18–5.43) | Middle Age Adults (45-64) | 2023 | 4.39 (4.25–4.53) | Older Adults (65+) | 2023 | 1.88 (1.77–1.99) |
